# Analysis of theoretical knowledge and the practice of science among brazilian otorhinolaryngologists

**DOI:** 10.5935/1808-8694.20130087

**Published:** 2015-10-08

**Authors:** Vitor Rosa Ramos de Mendonça, Thiago Alcântara, Nilvano Andrade, Bruno Bezerril Andrade, Manoel Barral-Netto, Viviane Boaventura

**Affiliations:** a PhD student.; b MD, ENT (MD, ENT, Santa Casa de Misericórdia da Bahia - Santa Izabel Hospital).; c MD, ENT, PhD (Coordinator of the Medical Residency Program, Santa Casa de Misericórdia da Bahia - Santa Izabel Hospital).; d MD, PhD (Clinical Research Fellow, Laboratory of Parasitic Diseases, National Institute of Allergy and Infectious Diseases, National Institutes of Health).; e MD, PhD (Full Researcher, Gonçalo Moniz Research Center - FIOCRUZ/BA; Full Professor, Department of Pathology, School of Medicine, Federal University of Bahia).; f MD, ENT, PhD (Preceptor, ENT Residency, Santa Izabel Hospital; Adjunct Professor, Department of Pathology, School of Medicine, Federal University of Bahia). Gonçalo Moniz Research Center - FIOCRUZ/BA and Federal University of Bahia.

**Keywords:** education, medical, knowledge, otolaryngology

## Abstract

Physicians from all medical specialties are required to understand the principles of science and to interpret medical literature. Yet, the levels of theoretical and practical knowledge held by Brazilian otorhinolaryngologists has not been evaluated to date.

**Objective:**

To assess the background and level of scientific knowledge of Brazilian otorhinolaryngologists.

**Method:**

Participants of two national ENT meetings were invited to answer a questionnaire to assess scientific practice and knowledge.

**Results and Conclusion:**

This study included 73 medical doctors (52% otorhinolaryngologists and 38% residents) aged between 18 and 65 years. About two-thirds have been involved in some form of scientific activity during undergraduate education and/or reported to have written at least one scientific paper. Physicians who took part in research projects felt better prepared to interpret scientific papers and carry out research projects (*p* = 0.0103 and *p* = 0.0240, respectively). Respondents who claimed to have participated in research or to have written papers had higher scores on theoretical scientific concepts (*p* = 0.0101 and *p* = 0.0103, respectively). However, the overall rate of right answers on questions regarding scientific knowledge was 46.1%. Therefore, a deficiency was observed in the scientific education of Brazilian otorhinolaryngologists. Such deficiency may be mitigated through participation in research.

## INTRODUCTION

Knowledge of scientific method and literature interpretation play an important role in the professional training of any medical specialty. The number of publications has grown as a reflex of the generation of new knowledge to be incorporated to medical practice. In order to keep up with the most current practices and ensure proper delivery of care, physicians need to understand the process of science production, to review it critically, and to apply scientific information rationally^1^. Physicians with experience in science can correctly interpret the literature to choose the best therapies for their patients and participate in research efforts to further the development of new approaches, therapies, and disease prevention methods^1^.

The undergraduate level education provided to physicians in Brazil lacks scientific training. Most medical students in Brazil do not take part in scientific research. According to Oliveira et al.^2^, only 12% of the students of six Brazilian medical schools have carried out research as part of their undergraduate studies. Involvement with research encourages medical students to follow careers in science and to carry out graduate level research^3^.

There is very little information on the type and quality of research training provided during medical specialization. In a family medicine residency program, the residents who underwent training on research acknowledged the value of the instruction they received for the therapy decision-making process^4^. However, only a small portion of the residents consider taking up a career on research or going to graduate school^5^.

The literature features no publications on the quality of ENT training in Brazil, despite the importance of scientific experience and knowledge for the practice of medicine^1^. This study aimed to assess the theoretical and practical knowledge of scientific research of ENT residents and physicians.

## METHOD

### Study design and participants

This is a cross-sectional study on the knowledge and practice of science among ENT physicians and residents in Brazil. The questionnaire used by Reis-Filho et al.^6^ on undergraduate students of Medicine and Law was adapted for physicians specialized on ENT care. The questions on scientific knowledge and practice were kept, and questions on workplace, time since graduation, and on whether the respondent was a physician or a resident were added. Participants of two national otorhinolaryngology meetings held in 2009 and 2010 were randomly invited to join the study and answer the questionnaire. The questionnaires were answered by the respondents as they visited our booth at the meetings. Volunteers were asked to answer the questionnaire only once. All questions had to be answered for the questionnaire to be included in the study. Respondents were not asked to identify themselves. The study was approved by the Research Ethics Committee (permit 361/2011).

### The questionnaire

Participants were asked to answer questions (Annex 1) on age, gender, and participation in undergraduate research projects, in addition to six multiple choice questions covering basic concepts of scientific method, statistics, and the structure of a scientific paper^6^. Questions to assess the respondents’ ability to interpret and write scientific papers, and to plan and conduct research projects were also included.

### Data analysis

Responses were categorized based on previous participation in undergraduate research projects, difference in theoretical scientific knowledge and subjective assessment in relation to previously written papers, and time since graduation. The multiple choice questions were expressed as a percentage of right answers. Differences were analyzed using Fisher's exact test. The Mann-Whitney test was used to calculate the mean theoretical performance of each group based on the mean number of right answers per individual in the six multiple choice theoretical questions. Software program GraphPad Prism^®^ 5.0 was used in data analysis. Statistical significance was attributed to events with *p* > 0.05.

## RESULTS

### Participant profile

Seventy-three physicians were enrolled in the study. Most were males (62.5%; n = 45), aged between 26 and 35 years (62.9%; n = 44), and graduated for 10 years or less (66.7%; n = 48). About 52% (n = 38) were specialist ENT physicians and 38% (n = 28) were ENT residents. (Table 1). Most of the respondents participated in undergraduate research (76.5%; n = 52) and/or wrote at least one scientific paper (78.4%; n = 40) (Table 1).

**Table 1:** Profile of individuals included in the study.

	n (%)
Residents	28 (38.4)
Otorhinolaryngologists	38 (52)
Not specified	7 (9.6)

Age	

18-25	4 (5.7)
26-35	44 (62.9)
36-45	6 (8.6)
46-55	13 (18.6)
56-65	3 (4.3)

Gender	

Male	45 (62.5)
Female	27 (37.5)

Time since graduation (years)	

> 10	48 (66.7)
≥ 10	24 (33.3)

Participation in research projects	

Yes	52 (76.5)
No	16 (23.5)

Authored scientific paper	

Yes	40 (78.4)
No	11 (21.6)

### Science theoretical concepts

**Figure 1:**
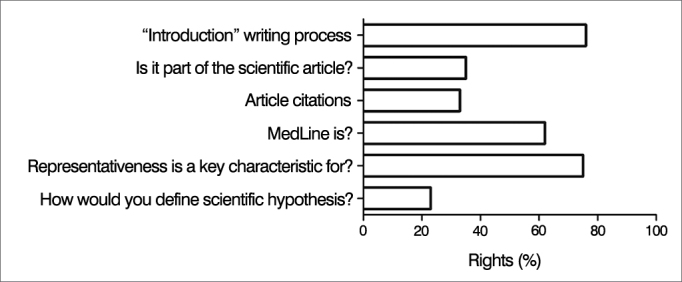
Percent rate of right answers for questions on the theory of scientific research. Overall performance of study participants in questions on the theory of scientific research. Values shown as percentage ratios of right answers for each question.

A mean of 46.1% of the responses to the six multiple choice questions designed to assess general concepts pertaining to research and applied sciences were right. Only 21.92% (n = 16) of the respondents answered correctly the question on the definition of scientific hypothesis, whereas about a third knew how to cite references (31.08%; n = 23) and how a scientific paper is structured (32.43%; n = 24) (Figure 1). Most participants gave right answers on the process of writing the introduction to a paper, on the MedLine grading system, and on the categorization of representativeness (Figure 1).

### Participation in research or writing of scientific paper

**Figure 2:**
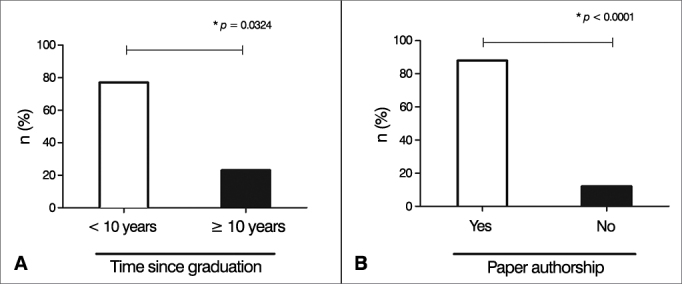
Association between time since graduation, paper authorship, and participation in research projects. Values are shown as absolute numbers of individuals who participated in research projects based on (A) time since graduation (> 10 years; ≥ 10 years) and (B) paper authorship. Fisher's exact test was used in both graphs to establish statistical differences.

**Figure 3:**
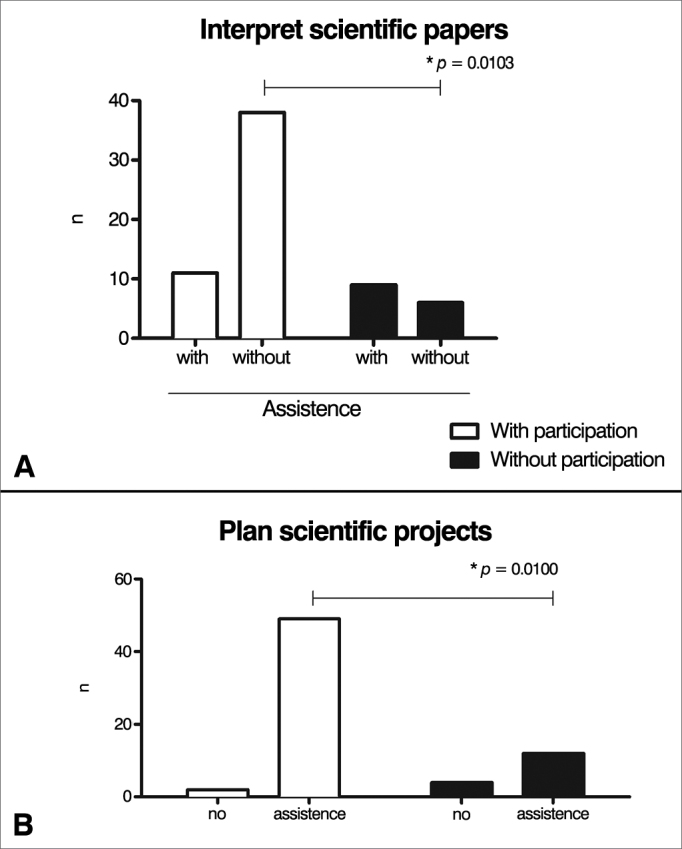
Impact of participation in research in the ability to interpret papers or plan scientific projects. Values are shown as absolute numbers of individuals. White bars represent individuals with prior participation in research projects; black bars show subjects without prior participation in research. Fisher's exact test was used in both graphs to establish statistical differences.

The ENT physicians and residents who participated in research projects graduated more recently (< 10 years) and had written at least one paper (*p* = 0.0324 and *p* < 0.0001, respectively) (Figure 2). Additionally, participants with previous research project experience felt more able to interpret scientific papers without assistance (*p* = 0.01) and to plan scientific projects with assistance (*p* = 0.02) (Figure 3).

**Figure 4:**
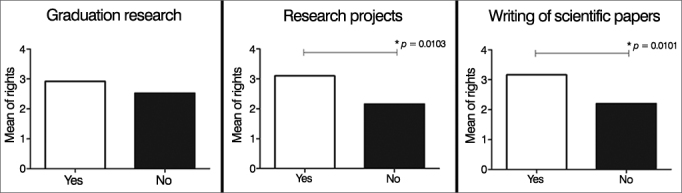
Mean theoretical performance according to participation in research, research project, and paper authorship. Values are shown as the mean number of right answers for each individual in the six multiple choice questions in the questionnaire. The Mann-Whitney test was used to establish the statistical differences in the graphs; only significant *p*-values are shown (*p* > 0.05).

**Figure 5:**
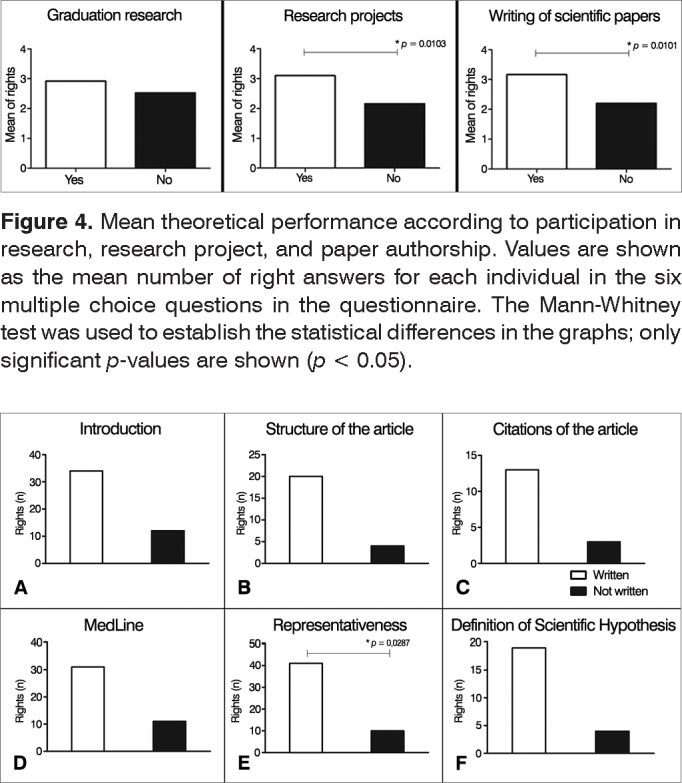
Performance on theoretical questions according to paper authorship. Values are shown as absolute numbers of individuals who answered each question correctly. White bars represent individuals who authored papers; black bars represent individuals who have not written papers. Fisher's exact test was used in the graphs to establish statistical differences. Only significant *p*-values were shown (*p* > 0.05).

Although 76.47% of the participants had taken part in undergraduate research and 78.4% had written scientific papers, the level of scientific theoretical knowledge among ENT physicians and residents was low (mean of 46.1% of right answers). The mean percentage of right answers to the six theoretical questions given by individuals who had participated in research projects was greater than the percentage seen in subjects without exposure to undergraduate research (3.1 vs. 2.16, *p* = 0.0103); the same was seen in regards to the writing of papers (3.2 vs. 2.2; *p* = 0.0101) (Figure 4). In the latter case, the performance of individuals who claimed to have written papers was better only on the question on “representativeness” (*p* = 0.0287), which raised the mean score of this group (Figure 5). On the other hand, involvement with undergraduate research (work as a research monitor, participation in scientific initiation projects) did not improve performance on questions on the theory of science (Figure 4).

## DISCUSSION

This study looked into the variations in quality of training on research by analyzing participants based on how long ago they graduated from medical school. Participation in undergraduate research projects was greater in the group of individuals graduated within ten years or less, showing improved access to scientific work. However, the scores of this group in answering theoretical questions were similar to the performance produced by physicians graduated for more than ten years.

Only participation in research projects was correlated with higher scores in questions on the theory of scientific knowledge. Individuals with prior experience on research also felt more capable of interpreting scientific papers without assistance and conducting research with assistance. Brazilian ENT physicians need to increase their participation in research groups in order to improve the quality of the scientific knowledge in the area. In addition to improving the scientific knowledge held by physicians, participation in research projects also encourages engagement in academic activity and promotes the training of new researchers.

Neacy et al.^7^ reported that residents who completed original undergraduate research projects were more interested in pursuing academic careers. Individuals engaged in undergraduate research published more papers (mean of four papers) than subjects not involved with research (mean of one paper) within the first 14 years of graduation^8^. The promotion of undergraduate research may meet the need for physician scientists and help developing countries become self-sufficient in the area of health research^9^.

Participants of research projects also had more experience writing scientific papers. Nonetheless, writing papers was not correlated with better scores on questions on the theory of scientific knowledge. The scores from theoretical questions seem to contradict history of publication, as this group was expected to know more about the structure of scientific papers. This study did not look into the type (original paper, review, case report, etc) or quality of the publications as possible explanations for the low scores attained in theoretical questions.

Despite the increase observed in the number of Brazilian publications on ENT in the last decade, from approximately 140 in 2000 to 260 in 2010, the number of citations is still low when compared to other countries (mean of 4.9 citations a year for Brazilian papers versus 13 in Thailand and 7.5 in Turkey, for example) or even to Brazilian publications from other medical specialties (mean of 7.4 citations per paper in ophthalmology and 10 in urology, for example)^10^. Greater participation in research projects may change this scenario and lead to improvements on the quality of the publications.

The low scores on scientific knowledge questions seen in this study may be the outcome of deficient training on research provided during medical school and extending into residency. In order to strengthen the presence of science work in undergraduate school, the curriculum of residency programs should contemplate research activities. Research during residency may improve clinical care by encouraging the development of reviewing skills, clinical rationale, and ongoing learning^11^, ^12^.

Program curricula in which research has been included were found to have residents with greater appreciation for research and more comfortable leading research projects^4^. Success factors for training on research include availability of guidance and coaching, training on basic research method, time and infrastructure for scientific research support^13^, ^14^, ^15^. In residency programs, science education includes lectures and seminars on the concepts of science, research projects, and paper presentations^16^.

The training of otorhinolaryngologist researchers, particularly through medical residency, was discussed in an ENT meeting in the United States^17^. It was suggested that more flexible programs and ongoing support during clinical training through faculty development are required to encourage otorhinolaryngologists to choose careers that combine research and clinical practice^17^. The results of this study would be better comprehended if objective data on the quality of research in residency programs in Brazil was available.

Extension and enhancement of the participation of residents in research on ENT may result in improved theoretical scientific knowledge and consequently better publications on otorhinolaryngology.

## CONCLUSIONS

Proper scientific knowledge plays a key role in the excellence of specialized medical care. Low scores on questions on the theory of scientific research were observed in this study. Scores were higher among individuals with prior participation in research projects, revealing the relevance of such experience in the basic scientific training of otorhinolaryngologists. Subjects with research experience claimed to be more able to interpret papers and conduct research projects. More investments on scientific education during the course of medical residency may change the scenario by enriching the education of Brazilian otorhinolaryngologists and contributing to the training of new physician researchers.

## Figures and Tables

**Table:** 

Specialist Physicians and Scientific Research

This questionnaire was designed to probe into the knowledge of basic concepts of science and research held by otorhinolaryngologists. If you wish to participate, please answer the questions below. The answers in this questionnaire will be used as basis for scientific and administrative discussion on the topic. The information provided herein is confidential. Respondents are not required to identify themselves. Thank you for your participation.

( ) ENT/Cardiologist ( ) Other medical specialty ( ) Resident

Workplace: (select one or more options)

( ) Private practice ( ) Public service ( ) Teaching hospital ( ) Private hospital ( ) Others:

Age: ( ) 18-25 years ( ) 26-35 years ( ) 36-45 years ( ) 46-55 years ( ) 56-65 years ( ) > 65 years

Gender: ( ) Male ( ) Female

Time since graduation: ( ) 5 years or less ( ) 5-10 years ( ) 10-20 years ( ) > 20 years

Were you involved in research while you were at medical school? ( ) Yes ( ) No If yes, name the activities based on the options below: ( ) Undergraduate research with scholarship. Time spent in activity: ( ) < 6 months ( ) 6 months-1 year ( ) > 1 year ( ) Course monitor Time spent in activity: ( ) < 6 months ( ) 6 months-1 year ( ) > 1 year ( ) Other: ______________________________

Please choose only one answer in the questions below.

Q1) How would you define SCIENTIFIC HYPOTHESIS?

( ) A proposed idea or thought ( ) An answer or solution to a problem ( ) An answer or solution to a problem that can be verified or demonstrated empirically ( ) Logic deduction of assumptions that may or not be verified empirically

Q2) Representativeness is a key characteristic of:

( ) Scientific papers with original data ( ) Meta-analysis papers ( ) Scientific research ( ) Sample

Q3) MedLine is:

( ) The first and better known online medical journal ( ) International Association of Medical Information Technology ( ) The printed version of the Medical Encyclopedia of scientific papers ( ) Acronym used to number the parts of the searched paper ( ) Medical database

Q4) Last year you published a paper on a relevant scientific journal. Now you wish to find how many times your paper has been cited. The best way to do that is through:

( ) Author index in the MedLine database ( ) Corporate index in the Science Citation Index database ( ) Author index in the Current Contents database ( ) Citation index in the Science Citation Index database ( ) Author index in the Science Citation Index database

Q5) A scientific paper contains:

( ) The author's CV ( ) Letter to the editor sent with the paper ( ) Description of schedule ( ) Acknowledgements to people who assisted in the production of the paper

Q6) All rules below apply to the process of writing an INTRODUCTION in a paper, EXCEPT FOR:

( ) Indicate clearly why the study was initiated ( ) Do not explain book data ( ) Do not explain the words from the title of the paper ( ) Make it long instead of concise ( ) Define clearly the question the study aims to answer

Q7) Can you interpret a scientific paper?

( ) No ( ) Yes, with assistance ( ) Yes, without assistance

Q8) Are you able to write a scientific paper?

( ) No ( ) Yes, with assistance ( ) Yes, without assistance

Q9) Have you participated in research projects?

( ) Yes ( ) No

Q10) Have you written a scientific paper?

( ) Yes ( ) No

Q11) Are you able to plan a scientific project?

( ) No ( ) Yes, with assistance ( ) Yes, without assistance

Q12) Are you able to conduct a scientific research project?

( ) No ( ) Yes, with assistance ( ) Yes, without assistance

Q13) Do you think otorhinolaryngologists have knowledge on scientific method?

( ) No ( ) Yes, with assistance ( ) Yes, without assistance

